# Proximity ligation assay to study TSH receptor homodimerization and crosstalk with IGF-1 receptors in human thyroid cells

**DOI:** 10.3389/fendo.2022.989626

**Published:** 2022-09-29

**Authors:** Christine C. Krieger, Alisa Boutin, Susanne Neumann, Marvin C. Gershengorn

**Affiliations:** Laboratory of Endocrinology and Receptor Biology, National Institute of Diabetes and Digestive and Kidney Diseases, National Institutes of Health, Bethesda, Maryland, United States

**Keywords:** Proximity Ligation Assay, PLA, homodimerization, protomer, monomer, receptor crosstalk, TSHR, IGF1R

## Abstract

Proximity ligation assay (PLA) is a methodology that permits detection of protein-protein closeness, that is, proteins that are within 40 nanometers of each other, in cells or tissues at endogenous protein levels or after exogenous overexpression. It detects the protein(s) with high sensitivity and specificity because it employs a DNA hybridization step followed by DNA amplification. PLA has been used successfully with many types of proteins. In this methods paper, we will describe the workings of PLA and provide examples of its use to study TSH/IGF-1 receptor crosstalk in Graves’ orbital fibroblasts (GOFs) and TSH receptor homodimerization in primary cultures of human thyrocytes.

## Introduction

**Figure 1:**
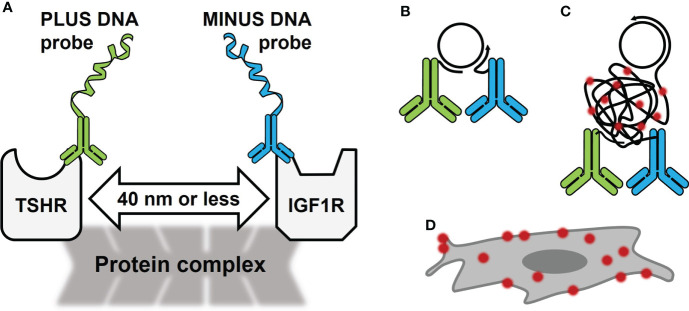
Schematic of Proximity Ligation Assay (PLA) in the TSHR/IGF1R Signalosome. **(A)** PLA oligonucleotides PLUS and MINUS are directly conjugated to primary antibodies for TSHR and IGF1R, respectively. These antibodies bind to the receptors’ extracellular regions on fixed cells. In a signalosome, the receptors were shown to be within 40 nm of each other. **(B)** PLUS and MINUS DNA probes will ligate and create a DNA circle that becomes a template for rolling circle amplification. **(C)** During amplification, fluorescently tagged oligonucleotides bind DNA. **(D)** PLA signals look like bright dots on the cell surface.

Proximity ligation assay (PLA) (1) is a methodology that permits detection of protein-protein closeness in cells or tissues at endogenous protein levels or after exogenous overexpression (2). In order for the protein(s) to be detected they must be within 40 nanometers of each other (**Figure 1A**). PLA utilizes specific DNA sequences covalently linked to antibodies specific for the protein(s) of interest (**Figure 1A**). It detects the protein(s) with high sensitivity and specificity because it employs a DNA hybridization step followed by DNA amplification with fluorescent probes (**Figure 1B, C**) to visualize interacting proteins by fluorescence microscopy (**Figure 1D**). Several forms of PLA have been developed (3, 4), but this review will focus on *in situ* PLA done on fixed cell samples. This type of PLA has been used successfully with many types of proteins (5) including G protein-coupled receptors and receptor tyrosine kinases (6, 7).

*In situ* PLA has some advantages over methods such as co-immunoprecipitation (Co-IP). Both PLA and Co-IP depend on antibodies that recognize proteins in the complex. Co-IP is a technique used to identify protein-protein interactions by using target protein-specific antibodies to capture proteins that are bound to a specific target protein. Unlike PLA, Co-IP occurs in solution. An antibody pulls down one protein member and its binding partners. These binding partners are later identified with Western blotting. For this method to work, interactions between proteins in the complex must be strong enough so that their affinity is maintained while in solution. Transient, low-affinity interactions, which are common in GPCR signaling, are difficult to detect with Co-IP. In contrast, PLA is done after fixation, which could capture transient events and preserve low-affinity interactions. Since this method uses fluorescent microscopy, information on subcellular location can also be collected. Compared to Co-IP, PLA can be scaled down in terms of number of cells and amount of reagents. For these reasons, PLA is better suited for studies of endogenously expressed proteins in primary cells.

To our knowledge, except for our studies, PLA has only been used in the field of thyroid research to show dimerization of the sodium-iodide symporter (NIS). It was used in experiments in SW1736 human anaplastic thyroid and HeLa human cervical cancer cell lines that were made to overexpress NIS (8). Since overexpression can be associated with aberrant protein localization and interactions (9), it is often better to study endogenously expressed proteins.

We elected to study potential interactions of TSH receptors (TSHRs) and IGF-1 receptors (IGF1Rs) in primary cultures of human thyroid cells and of human orbital fibroblasts/pre-adipocytes (OFs) as those play a pivotal role in the pathogenesis of Graves’ disease and associated thyroid eye disease. We were able to study these receptor interactions primarily using endogenously expressed receptors and used overexpression of TSHR to complement these findings. Specifically, we studied TSHR homodimerization and TSHR/IGF1R crosstalk. In the following sections, we will briefly review how PLA was used to extend our understanding of TSHR signaling and describe considerations that were taken into account when adapting PLA to our specific experimental systems. We will then detail the procedures used to detect receptor closeness using *in situ* Duolink PLA.

## TSHR/IGF1R crosstalk

The involvement of TSHRs and IGF1Rs acting together to regulate responses in thyrocytes and OFs has been well-documented (10). To summarize what has been recently reviewed (11, 12), IGF1R inhibitors may partially inhibit TSHR signaling (13–15); simultaneous activation of TSH and IGF1 receptors can lead to greater-than-additive downstream signals (16, 17); and some TSHR stimulating antibodies may initiate both IGF1R-independent and -dependent pathways (18). However, the molecular details of the apparent synergy that mediates this effect is not fully elucidated. Various models of receptor crosstalk have been described (19, 20), and not all require receptors to be proximal to each other. Therefore, PLA can be used to narrow down which model most likely applies to the disease being studied.

**Figure 2:**
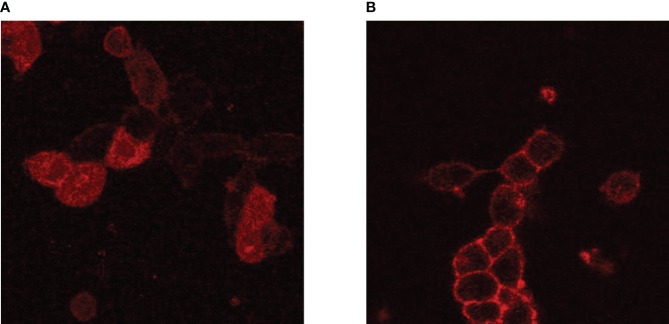
Comparison of IGF1R antibodies in fluorescent microscopy. HEK-TSHR cells were fixed with 4% paraformaldehyde and incubated with 5 µg/mL IGF1R antibodies overnight at 4°C. An anti-mouse-Alexa-647 secondary antibody was used at a 1:20,000 dilution for 30 minutes at room temperature. IGF1R (red) were detected using IGF1R antibody clone 1H7 **(A)** or clone αIR3 **(B)**. Even though both 1H7 and αIR3 recognize IGF1R in live cells, cell-fixation may change the antibody epitope on IGF1R, results in non-specific binding of 1H7. This did not occur for αIR3, which showed membrane-specific fluorescence. However, this cannot be predicted, and every antibody must be verified prior to experiments.

Adapting PLA to study receptor signaling required additional considerations for antibody selection. Unlike experiments with proteins in solution, the spatial orientation of receptors within the plasma membrane remains intact. Therefore, the antibody epitopes must either be both in the cytoplasmic side or the extracellular side. Even if cytoplasmic and extracellular epitopes are closer than 40 nm apart, PLA probes cannot cross the plasma membrane. Notably, PLA experiments in our lab using IGF1R antibodies that bind to the α and β subunits were unsuccessful (unpublished) despite the fact that homodimer formation is an integral characteristic of IGF1R. At the time this was written, a majority of commercially available antibodies for TSHR have epitopes on the extracellular domain, and several IGF1R antibodies for the α-subunit are available. However, the antibodies that were validated for flow cytometry are not guaranteed to work for microscopy. For example, 1H7, an IGF1R antibody, could recognize IGF1R in live cells during flow cytometry (21) but produced weak membrane fluorescence in fixed cells that we interpret as non-specific (**Figure 2A**). In contrast, the plasma membrane-localized fluorescence detected with αIR3, another mouse monoclonal anti-IGF1R antibody, was strong in fixed cells (**Figure 2B**). Because of the limited antibody selection, covalent conjugation of PLUS and MINUS PLA probes is practically a requirement^1^. TSHR antibodies are almost exclusively human or mouse, as are antibodies for the IGF1R α-subunit. The few rabbit IGF1R α-subunit antibodies were excluded because they did not pass flow cytometry or Duolink single-recognition control experiments. As a result, only a handful of TSHR and IGF1R antibodies are suitable for PLA studies.

**Figure 3:**
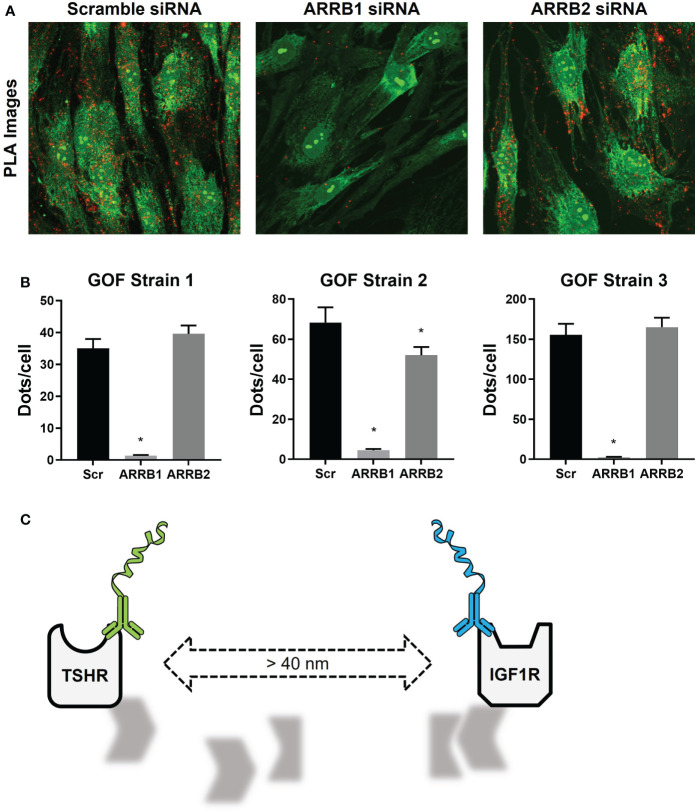
TSHR/IGF1R Signalosomes in Human Graves’ Orbital Fibroblasts. **(A)** Duolink PLUS and MINUS probes were conjugated to TSHR antibody KSAb1 and IGF1R antibody αIR3, respectively. TSHR/IGF1R proximity was shown in fixed GOFs treated with Scramble, β-arrestin 1 (ARRB1), or β-arrestin 2 (ARRB2) siRNA. Duolink signals are shown in red. Nuclear and cell staining with SYTO-9 is shown in green. **(B)** Quantification of positive PLA signals (Dots) per cell are shown for three GOF strains. ARRB1 siRNA significantly reduced the number of positive PLA signals in all strains, whereas reduction by ARRB2 siRNA was only significant in one. *indicates P < 0.05. **(C)** In the absence of β-arrestin 1, the signalosome dissociates, increasing the distance between TSHR and IGF1R to greater than 40 nm, leading to a loss of PLA signals. Panels **(A, B)** were adapted with permission from Krieger et al. (10).

In our experiments with thyrocytes and OFs, PLA was used to not only show that TSHR and IGF1R were physically close to each other, but also to demonstrate a role for beta-arrestin 1 as a scaffolding protein in this mechanism. Beta-arrestin 1 was necessary to the cohesion of the scaffold required to maintain close proximity of TSHR and IGF1R (**Figure 3A, B**) (22). When that closeness was disrupted by beta-arrestin 1 knockdown TSHR/IGF1R crosstalk was prevented (**Figure 3C**) (22).

PLA is a promising tool to investigate the remaining questions for TSHR/IGF1R crosstalk. The above studies have only identified one member of the TSHR/IGF1R signalosome, β-arrestin 1. While this protein has been shown to bind individually to TSHR or IGF1R, it is also a scaffold for several other proteins. TSHR and IGF1R are also known to form complexes, and the likelihood other proteins are present in the signalosome are high. Although experiments are limited by the performance of available antibodies, the payoff for these studies offset technical hurdles.

## TSHR homodimerization

TSHRs have previously been shown to homodimerize using functional (23) and biophysical assays (fluorescence and bioluminescence resonance energy transfer) (24). For the purposes of this methodology paper, we use the term monomer to describe individual TSHRs that are not associated with another TSHR; protomer to describe single TSHR within a complex of at least one other TSHRs; and homodimer to represent a dimer or a higher order oligomer of TSHR protomers. Homodimerization occurs during receptor biosynthesis and translocation to the cell-surface membrane (25). Homodimerization expands the biologic responses initiated by TSHR activation. For example, coupling to and activation of Gs proteins to stimulate the production of cAMP occurs at low TSH concentrations that allows binding to one protomer of the TSHR dimer whereas coupling to and activation of Gq/11 proteins to stimulate the production of inositol trisphosphate occurs at high TSH concentrations that allows binding to both protomers of TSHR dimers (26).

**Figure 4:**
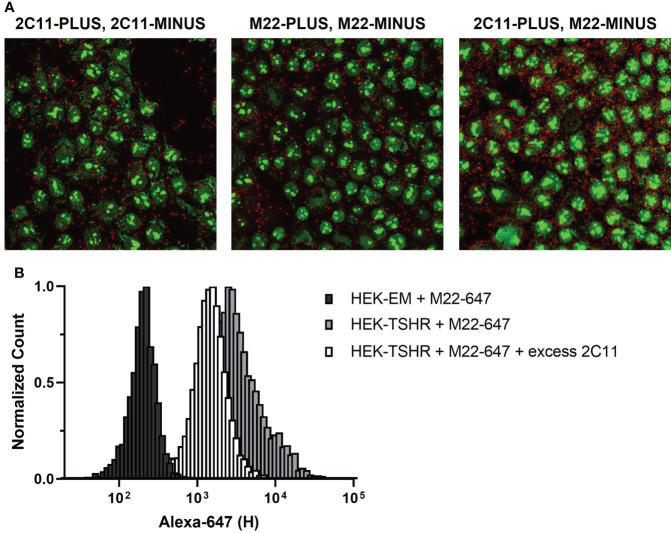
TSHR Duolink in HEK-TSHR. When PLUS and MINUS conjugates of the same monoclonal antibody are used in PLA, signals will only be produced when PLUS and MINUS conjugates bind to different protomers in a homodimer. We show this in fixed HEK-TSHR cells **(A)**. Anti-TSHR antibodies 2C11 and M22 were directly conjugated to PLUS and MINUS probes and used to detect TSHR. TSHR homodimers were detected with 2C11-PLUS/MINUS (left) and M22-PLUS/MINUS (middle). In contrast, the higher number of PLA signals detected using 2C11-PLUS and M22-MINUS are from both TSHR homodimers and individual TSHR (right). Since 2C11 and M22 bind to different epitopes on TSHR, it is possible for the two antibodies to bind simultaneously to the same receptor. This was supported experimentally using flow cytometry **(B)**. M22 was conjugated to Alexa Fluor 647 (M22-647) and used to detect TSHR in HEK-TSHR cells. M22-647 bound non-specifically to HEK parental cells (black bars), which do not express TSHR. In HEK-TSHR cells, binding of M22-647 right-shifted the flow cytometry peak (gray bars). An excess of 2C11 did not completely outcompete M22-647 binding (white bars). This is consistent with the idea that M22 and 2C11 may bind to the same TSHR protomer/monomer.

As with receptor crosstalk, optimizing PLA to investigate TSHR homodimerization requires careful selection of primary antibodies. A majority of commercially available anti-TSHR antibodies are monoclonal and bind to a single epitope in the extracellular domain, i.e. they can only form a single complex with a TSHR protomer. Thus, a Duolink signal between monoclonal TSHR antibodies could only occur between two protomers of a TSHR homodimer. This applies to common TSHR monoclonal antibodies 2C11 and M22 (**Figure 4A**, left and middle panels). Of note, PLA with a mixture of 2C11-PLUS and M22-MINUS resulted in more signals than using 2C11-PLUS/MINUS or M22-PLUS/MINUS (**Figure 4A**, right panel). This is most likely due to the fact that using the two antibodies may allow both antibodies to bind to monomeric TSHRs in addition to TSHR homodimers (**Figure 4B**).

**Figure 5:**
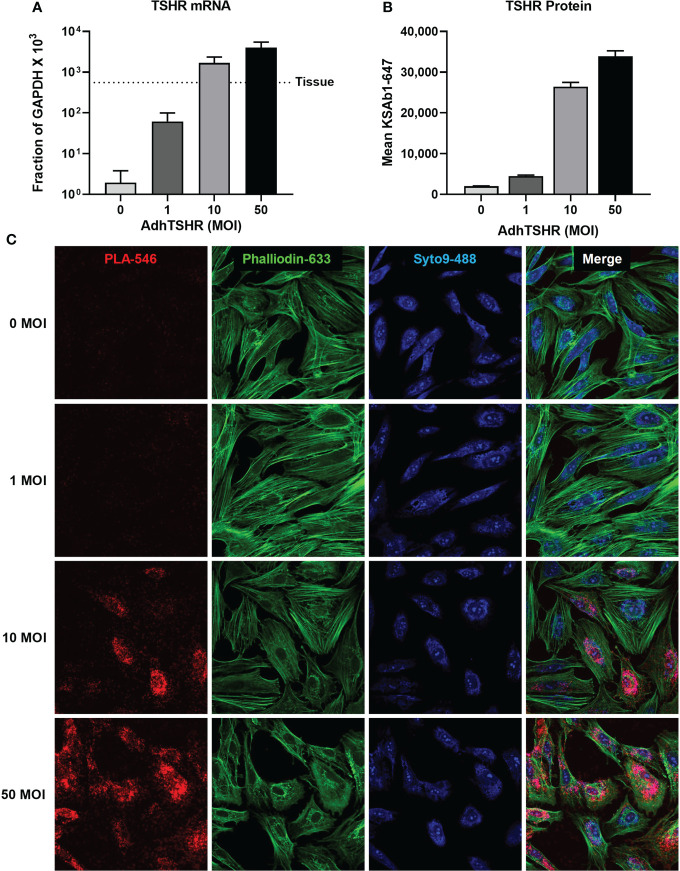
TSHR Homodimers in Human Thyrocytes. **(A)** Human thyrocytes were infected with adenovirus expressing full-length human TSHR (AdhTSHR) to approach mRNA expression levels measured in human thyroid tissue with increasing Multiplicity of Infection (MOI). **(B)** TSHR surface expression was measured on live thyrocytes using flow cytometry. Bars depict mean KSAb1-647 fluorescence of labeled cells. **(C)** TSHR homodimers were detected on fixed thyrocytes with PLA. Mouse anti-TSHR antibody KSAb1 was conjugated to PLUS and MINUS Duolink probes. Duolink signals are pseudo-colored in red. Phalloidin stain (green) determined cell area. Nuclei were stained with Syto9 (blue) and used to determine cell number. Figure reproduced with permission from Boutin et al. (27).

In our studies, *in situ* PLA allowed us to investigate aspects of homodimerization using primary cultures of thyrocytes, where downstream signaling pathways and regulation are more or less intact. Within this platform, we can probe questions that are masked in artificial systems. For example, in a previous study (27), we showed that TSHR mRNA in thyroid tissue was orders of magnitude greater than in cultures of primary thyrocytes (**Figure 5A**). We used Adenovirus expressing full-length human TSHR (AdhTSHR) to increase TSHR protein expression to levels similar to that of thyroid tissue (**Figure 5B**) and quantified the relative amounts of TSHR present as homodimers. KSAb1 was directly conjugated to PLUS and MINUS oligo probes and used to detect TSHR within 40 nm of each other, that is, within homodimers (**Figure 5C**). We determined that homodimerization increases with TSHR expression (**Figure 5C**) and demonstrated a phenomenon of hormetic cyclic AMP dose response to TSH (27). We proposed this mechanism could be negative feedback preventing thyroid overstimulation. Given that TSHR expression in thyroid tissue is orders of magnitude greater in thyrocyte cell culture (27), TSHR homodimerization and its subsequent signaling pathways could be more prevalent than previously appreciated.

## Protocol for visualizing of receptor closeness using *In Situ* Duolink PLA

### Materials

- MatTek dishes with No. 1.5 coverslip, 14 mm microwell diameter, poly-D-lysine coating (MatTek, P35GC-1.5-14-C)
- 4% methanol-free paraformaldehyde in PBS (Electron Microscopy Sciences, 157-4)
- Fine-tipped transfer pipets (Thermo Fisher, 233-20S)
- Sterile PBS
- Duolink *In Situ* Probemaker kits (Millipore Sigma, DUO92009 and DUO92010)
- Molecular Biology Grade Water (Quality Biological, 351-029-101)
* This is used to dilute all reagents*
- Blocking Solution
  • Duolink 1x Assay Reagent (Millipore Sigma, DUO92009 and DUO92010)
  • 0.3 M glycine
  • 1% normal goat serum (Millipore Sigma, NS02L)
  • 1x PBS
- PLA Probe Diluent (Millipore Sigma, DUO92009 and DUO92010)
- Heat block
- Duolink® Fluorescent Detection Reagent Orange (Millipore Sigma, DUO92007)
  • 5x Ligation Buffer
  • Ligase stock
  • 5x Amplification Buffer
  • Polymerase stock
- Wash Buffer A (Millipore Sigma, DUO82049)
- Wash Buffer B (Millipore Sigma, DUO82049)
- SYTO 9 green, fluorescent nucleic acid stain, 5mm in DMSO (Thermo Fisher, S34854)
- Alexa Fluor™ 647 Phalloidin (Thermo Fisher, A22287), 200 U/mL in methanol

### Sample preparation

1. If necessary, concentrate or purify antibody to 1 mg/mL in PBS. A minimum of 20 µL of antibody solution is necessary. Begin antibody conjugation to PLUS or MINUS oligonucleotide probes following directions in the Duolink Probemaker kits.

Direct conjugation of PLUS and MINUS oligos is required for human TSHR antibodies such as M22 and strongly suggested for TSHR antibodies from other species.

2. Plate cells in the poly-D-lysine-coated microwell of a 35mm MatTek dish. The 14mm microwell typically holds 150 – 200 µL of cell suspension. Evaporation is not an issue if the MatTek dishes are kept in a humid incubator for 24 hours. For longer incubation times, the entire 35 mm dish can be filled with 2 mL of media after the cells have adhered to the coverslip. Alternatively, drops of sterile water can be pipetted along the edge of the MatTek.

Cells should be plated at 70% confluence. If cell density is too high, receptor epitopes may not adequately be exposed to detection antibodies or reagents. However, plating primary orbital fibroblasts or thyrocytes too sparsely diminishes cell viability.

We have found that MatTek dishes perform better in PLA compared to chambered coverglass. In the latter Duolink assay, the wells in the chambered coverglass have a strong capillary effect, and a greater volume of reagent solution is required to keep fixed sample immersed during incubations.

3. Following treatments and incubations, aspirate media and rinse microwells 3x with 150 µL of ice-cold PBS. Immediately fix cells with 150 µL of 4% formaldehyde in PBS for 10 – 15 minutes at room temperature. Remove fixative with 3x rinses with room temperature PBS. Fixed cells may be stored in PBS at 4°C.

### Duolink assay

4. Preheat plate warmer to 37°C and prepare blocking solution (above). The addition of glycine to the blocking solution greatly diminishes background caused by formaldehyde fixation. If 20x Duolink Assay Reagent is not available, sequentially blocking with 0.3 M glycine in PBS for 30 minutes at room temperature, then Duolink Blocking Buffer (found in Duolink Detection Reagent Kit) for 1 hour at 37°C is an alternative.

Aspirate PBS from dish and if necessary, use a Kimwipe to dry the plastic portion of the dish bottom, taking care to keep the microwell moist. Pipet approximately 100 µL of blocking solution into the microwells of each MatTek dish. 80 µL is minimum volume for a 14 mm microwell. Incubate MatTek dishes for 1 hour on the plate warmer pre-heated to at 37°C. Surround the MatTek dish with wet Kimwipes to provide a humidified environment.

It is essential that the coverslip be in direct contact with a pre-warmed metallic block. Heat transfer from the shelves typically found in incubators is less efficient, resulting poorer PLA signal.

5. Dilute PLUS and MINUS conjugated antibodies in PLA Probe Diluent. In our studies, final concentrations of 4 µg/mL of PLUS antibody and 4 µg/mL of MINUS antibody were sufficient. Using a fine-tipped transfer pipet, aspirate the blocking solution and add 150 – 200 µL of antibody solution to the microwells. In the no antibody control, add 150 – 200 µL of PLA Probe Diluent. Incubate dishes in a humidified chamber overnight at 4°C.

Aspirating liquids with vacuum suction may lead to cell loss on the coverslip. Using a transfer pipet will minimize this.

6. Thaw 5x Duolink Ligation and Amplification stocks and prepare 1x solutions using Molecular Biology Grade water. The following steps will require 80 µL of solution per microwell. Keep Ligase and Polymerase stocks in benchtop freezer block.

7. Pre-heat plate warmer and wash cells 2x for 5 minutes in 80 µL 1x Wash Buffer A. During the second rinse, prepare Ligase solution by diluting the 1 U/µL Ligase stock 1:40 in Ligation Buffer. Aspirate Wash Buffer A and add 80 µL of Ligase solution to each microwell. Place dishes on a plate warmer and line edges of plate warmer with wet Kimwipes. Incubate cells with Ligase for 30 minutes at 37°C.

8. Wash cells 2x for 5 minutes with 1x Wash Buffer A. During second wash, dilute the 10 U/µL Polymerase stock 1:80 in Amplification buffer. Aspirate Wash Buffer A and add 80 µL of Amplification solution to each microwell. Place dishes on plate warmer, rewet Kimwipes if necessary, and incubate cells at 37°C for 1 hour and 40 minutes.

9. Aspirate Amplification solution with a fine-tipped transfer pipet and rinse cells with 1x Wash Buffer B for 10 minutes 2x at room temperature. Meanwhile, dilute SYTO 9 stock and Alexa Fluor 647 Phalloidin stock 1:10,000 in 1x Wash Buffer B. Aspirate microwells and add SYTO 9/Phalloidin solution. Incubate 10 minutes at room temperature. Note, this adds a third wash in Wash Buffer B. Lower concentrations of SYTO 9 and phalloidin can be used with increased incubation time.

10. Dilute Wash Buffer B 1:100 in water. Wash cells in 0.01x Wash Buffer B for 1 minute.

11. Aspirate wells and flood entire 35 mm dish with 2 mL of PBS and proceed with image acquisition as described below.

The Duolink signal lasts approximately one week under these conditions.

### Microscopy and image analysis

12. On an LSM 510 Meta Inverted Confocal microscope, add one drop of Zeiss Immersol Immersion oil to the Plan-neofluar 40x/1.3 Oil DIC objective

13. Insert the MatTek dish with the secondary-only control into a stage adaptor for a 35 mm dish and find the focal plane using the eyepiece and transilluminated light.

14. Set software to Multi Track mode and apply the appropriate filter sets for the 488 and 543 lasers. Set the pinhole to 1 Airy unit, and in a secondary-only control sample, empirically determine the Gain.

The fluorescence in the 488 channel is not quantified, so the Gain can be adjusted so that the nuclei are distinguishable. In adherent, eukaryotic cells, SYTO 9 diffuse cytoplasmic staining in addition to nuclear staining may occur. Despite this, cell nuclei are readily quantified.

The Duolink signal will be quantified in the 543 channel. It is important keep the Gain and other microscope settings constant amongst the different samples. With the secondary-only control, set the Gain so that non-specific fluorescence is barely visible. This will ensure that signal above this is specific.

15. If it is necessary to increase the Gain past 750, consider increasing laser power. It may also be necessary to troubleshoot the PLA assay. Switch the secondary-only control sample with an experimental sample and find the focal plane using the eyepiece. It may be necessary to add an additional drop of oil on the objective.

16. To acquire a Z Stack, use the 488 channel only to mark the First and Last positions. In the interest of time, the number of slices may be adjusted, however the interval between slices must be constant from sample to sample. An interval of 1 µm or smaller is recommended.

17. After the First and Last positions of the Z Stack have been set, turn on both the 488 and 543 channels and start image acquisition.

If the Duolink signal saturates the 543 channel, acquisition may be stopped and the Gain can be lowered. However, this new Gain must be the same between experimental samples.

18. After image acquisition, download the images in lsm format and open with ImageJ.

19. In ImageJ, make a Z-projection using Max Intensity for the Syto 9 and Duolink channels in order to construct single 2-D images from the Z Stack. If Background Subtraction is done on the Syto 9 channel, then Background subtraction with the same Rolling Ball Radius must be done on all other Duolink images.

20. On the Syto 9 image, set the threshold so that all Duolink dots are selected. Record Threshold number in order to apply the same value when analyzing the other Duolink images.

21. Analyze Particles using the built-in ImageJ algorithm. For our GOFs and thyrocytes, we set the minimum particle size to 5 pixels. This may change based on the experimental system. Record the number of particles, this is the number of Duolink signals in the image.

22. Switch to the Syto 9 Z-projection and count the number of nuclei using ImageJ’s Multi-point Tool. Record the number of nuclei and divide number of particles by number of nuclei to calculate dots/cell.

In some images, cells on the edge may be cut off, and the total number of dots on that cell are not recorded. It is therefore necessary to image greater than 50 cells so that these edge effects average out.

## Data availability statement

The original contributions presented in the study are included in the article/supplementary material. Further inquiries can be directed to the corresponding author.

## Author contributions

CK contributed original experimental data. MG, SN, CK, and AB wrote the manuscript. All authors agree to be accountable for the content of the work. All authors contributed to the article and approved the submitted version.

## Funding

This work was supported by the Intramural Research Program of the National Institute of Diabetes and Digestive and Kidney Diseases, National Institutes of Health (Z01 DK011006).

## Conflict of interest

MCG is a consultant with Crosstalk Therapeutics LTD.

The remaining authors declare that the research was conducted in the absence of any commercial or financial relationships that could be constructed as a potential conflict of interest.

## Publisher’s note

All claims expressed in this article are solely those of the authors and do not necessarily represent those of their affiliated organizations, or those of the publisher, the editors and the reviewers. Any product that may be evaluated in this article, or claim that may be made by its manufacturer, is not guaranteed or endorsed by the publisher.

## Footnotes

1 Duolink Anti-human-PLUS and anti-human-MINUS secondary antibodies are now commercially available. However, they have not been tested by our lab.

## References

[1] Fredriksson S Gullberg M Jarvius J Olsson C Pietras K Gustafsdottir SM . Protein detection using proximity-dependent DNA ligation assays. Nat Biotechnol (2002) 20(5):473–7. doi: 10.1038/nbt0502-473 11981560

[2] Alam MS . Proximity ligation assay (Pla). Curr Protoc Immunol (2018) 123(1):e58. doi: 10.1002/cpim.58 30238640 PMC6205916

[3] Leuchowius KJ Soderberg O Kamali-Moghaddam M Jarvius M Weibrecht I Pardali K . Protein diagnostics by proximity ligation: Combining multiple recognition and DNA amplification for improved protein analyses. In: Molecular diagnostics, 2nd Edition. Cambridge MA, USA: Academic Press (2010). p. 299–306. doi: 10.1016/B978-0-12-374537-8.00020-1

[4] Ullman EF . Homogeneous immunoassays. In: The immunoassay handbook. Amsterdam, Netherlands: Elsevier Science (2013). p. 67–87.

[5] Hegazy M Cohen-Barak E Koetsier JL Najor NA Arvanitis C Sprecher E . Proximity ligation assay for detecting protein-protein interactions and protein modifications in cells and tissues in situ. Curr Protoc Cell Biol (2020) 89(1):e115. doi: 10.1002/cpcb.115 33044803 PMC8041061

[6] Taura J Fernandez-Duenas V Ciruela F . Visualizing G protein-coupled receptor-receptor interactions in brain using proximity ligation in situ assay. Curr Protoc Cell Biol (2015) 67:17 1– 6. doi: 10.1002/0471143030.cb1717s67 26061241

[7] Faron-Gorecka A Szlachta M Kolasa M Solich J Gorecki A Kusmider M . Understanding gpcr dimerization. Methods Cell Biol (2019) 149:155–78. doi: 10.1016/bs.mcb.2018.08.005 30616817

[8] Thompson RJ Fletcher A Brookes K Nieto H Alshahrani MM Mueller JW . Dimerization of the Sodium/Iodide symporter. Thyroid (2019) 29(10):1485–98. doi: 10.1089/thy.2019.0034 PMC6797079 31310151

[9] Moriya H . Quantitative nature of overexpression experiments. Mol Biol Cell (2015) 26(22):3932–9. doi: 10.1091/mbc.E15-07-0512 PMC4710226 26543202

[10] Krieger CC Morgan SJ Neumann S Gershengorn MC . Thyroid stimulating hormone (Tsh)/Insulin-like growth factor 1 (Igf1) receptor cross-talk in human cells. Curr Opin Endocr Metab Res (2018) 2:29–33. doi: 10.1016/j.coemr.2018.01.007 30547142 PMC6287758

[11] Krieger CC Neumann S Gershengorn MC . Tsh/Igf1 receptor crosstalk: Mechanism and clinical implications. Pharmacol Ther (2020) 209:107502. doi: 10.1016/j.pharmthera.2020.107502 32061922 PMC7187798

[12] Smith TJ . Insulin-like growth factor pathway and the thyroid. Front Endocrinol (Lausanne) (2021) 12:653627. doi: 10.3389/fendo.2021.653627 34149612 PMC8212127

[13] Chen H Mester T Raychaudhuri N Kauh CY Gupta S Smith TJ . Teprotumumab, an igf-1r blocking monoclonal antibody inhibits tsh and igf-1 action in fibrocytes. J Clin Endocrinol Metab (2014) 99(9):E1635–40. doi: 10.1210/jc.2014-1580 PMC4154099 24878056

[14] Kumar S Iyer S Bauer H Coenen M Bahn RS . A stimulatory thyrotropin receptor antibody enhances hyaluronic acid synthesis in graves' orbital fibroblasts: Inhibition by an igf-I receptor blocking antibody. J Clin Endocrinol Metab (2012) 97(5):1681–7. doi: 10.1210/jc.2011-2890 PMC3339886 22399503

[15] Place RF Krieger CC Neumann S Gershengorn MC . Inhibiting Thyrotropin/Insulin-like growth factor 1 receptor crosstalk to treat graves' ophthalmopathy: Studies in orbital fibroblasts in vitro. Br J Pharmacol (2017) 174(4):328–40. doi: 10.1111/bph.13693 PMC5289943 27987211

[16] Krieger CC Gershengorn MC . A modified Elisa accurately measures secretion of high molecular weight hyaluronan (Ha) by graves' disease orbital cells. Endocrinology (2014) 155(2):627–34. doi: 10.1210/en.2013-1890 PMC3891933 24302624

[17] Morgan SJ Neumann S Marcus-Samuels B Gershengorn MC . Thyrotropin and insulin-like growth factor 1 receptor crosstalk upregulates sodium-iodide symporter expression in primary cultures of human thyrocytes. Thyroid (2016) 26(12):1794–803. doi: 10.1089/thy.2016.0323 PMC5175432 27638195

[18] Krieger CC Neumann S Place RF Marcus-Samuels B Gershengorn MC . Bidirectional tsh and igf-1 receptor cross talk mediates stimulation of hyaluronan secretion by graves' disease immunoglobins. J Clin Endocrinol Metab (2015) 100(3):1071–7. doi: 10.1210/jc.2014-3566 PMC4333041 25485727

[19] Pyne NJ Pyne S . Receptor tyrosine kinase-G-Protein-Coupled receptor signalling platforms: Out of the shadow? Trends Pharmacol Sci (2011) 32(8):443–50. doi: 10.1016/j.tips.2011.04.002 21612832

[20] Forrester SJ Kawai T O'Brien S Thomas W Harris RC Eguchi S . Epidermal growth factor receptor transactivation: Mechanisms, pathophysiology, and potential therapies in the cardiovascular system. Annu Rev Pharmacol Toxicol (2016) 56:627–53. doi: 10.1146/annurev-pharmtox-070115-095427 PMC5477061 26566153

[21] Jamitzky S Krueger AC Janneschuetz S Piepke S Kailayangiri S Spurny C . Insulin-like growth factor-1 receptor (Igf-1r) inhibition promotes expansion of human nk cells which maintain their potent antitumor activity against Ewing sarcoma cells. Pediatr Blood Cancer (2015) 62(11):1979–85. doi: 10.1002/pbc.25619 26131572

[22] Krieger CC Boutin A Jang D Morgan SJ Banga JP Kahaly GJ . Arrestin-Beta-1 physically scaffolds tsh and Igf1 receptors to enable crosstalk. Endocrinology (2019) 160(6):1468–79. doi: 10.1210/en.2019-00055 PMC6542485 31127272

[23] Urizar E Claeysen S Deupi X Govaerts C Costagliola S Vassart G . An activation switch in the rhodopsin family of G protein-coupled receptors: The thyrotropin receptor. J Biol Chem (2005) 280(17):17135–41. doi: 10.1074/jbc.M414678200 15722344

[24] Persani L Calebiro D Bonomi M . Technology insight: Modern methods to monitor protein-protein interactions reveal functional tsh receptor oligomerization. Nat Clin Pract Endocrinol Metab (2007) 3(2):180–90. doi: 10.1038/ncpendmet0401 17237844

[25] Drake MT Shenoy SK Lefkowitz RJ . Trafficking of G protein-coupled receptors. Circ Res (2006) 99(6):570–82. doi: 10.1161/01.RES.0000242563.47507.ce 16973913

[26] Allen MD Neumann S Gershengorn MC . Occupancy of both sites on the thyrotropin (Tsh) receptor dimer is necessary for phosphoinositide signaling. FASEB J (2011) 25(10):3687–94. doi: 10.1096/fj.11-188961 PMC3177577 21705666

[27] Boutin A Krieger CC Marcus-Samuels B Klubo-Gwiezdzinska J Neumann S Gershengorn MC . Tsh receptor homodimerization in regulation of camp production in human thyrocytes in vitro. Front Endocrinol (Lausanne) (2020) 11:276. doi: 10.3389/fendo.2020.00276 32425890 PMC7203478

